# Supplementary material for: Noncommunicable disease behavioral risk factors in Sub Saharan Africa: A protocol of systematic review and meta-analysis
Source: PLoS One. 2024 Oct 10;19(10):e0311322. doi: 10.1371/journal.pone.0311322 (PMC11466397; doi:10.1371/journal.pone.0311322)
Supplement: S2 File — (DOCX) [file pone.0311322.s002.docx]

**Proposed search strategy in PubMed**

1. "Non-communicable disease*"[ti]
2. "Noncommunicable disease*"[ti]
3. "Chronic disease*"[ti]
4. Neoplasm [MeSH]
5. Cancer* [ti]
6. carcinoma[ti]
7. Diabetes Mellitus[MeSH]
8. Diabetes[ti]
9. Cardiovascular Diseases [MeSH]
10. "Coronary heart disease"[ti]
11. "Cerebrovascular disease"[ti]
12. "Rheumatic heart disease"[ti]
13. Respiratory Tract Diseases [MeSH]
14. "Chronic Obstructive Lung Disease*"[ti]
15. "Chronic Obstructive Pulmonary Disease*"[ti]
16. COPD [ti]
17. COAD [ti]
18. Asthma*[ti]
19. "Bronchial asthma"[ti]
20. "Pulmonary hypertension"[ti]
21. Overweight [MeSH]
22. "Excess weight"[ti]
23. "Over weight"[ti]
24. Obesity[ti]
25. Hyperglycemia [MeSH]
26. "High blood glucose"[ti]
27. "Raised blood glucose"[ti]
28. "Blood sugar"[ti]
29. Hyperlipidemias [MeSH]
30. cholesterol[ti]
31. "Raised blood pressure"[ti]
32. Hypertension [MeSH]
33. "High blood pressure"[ti]
34. #1 OR #2 OR #3 OR #4 OR #5 OR #6 OR #7 OR #8 OR #9 OR #10 OR #11 OR #12 OR #13 OR #14 OR #15 OR #16 OR #17 OR #18 OR #19 OR #20 OR #21 OR #22 OR #23 OR #24 OR #25 OR #26 OR #27 OR #28 OR #29 OR #30 OR #31 OR #32 OR #33
35. Risk factor*[ti]
36. "Associated factor*"[ti]
37. Predictor*[ti]
38. Determinant*[ti]
39. "Life style*"[ti]
40. Lifestyle*[ti]
41. #35 OR #36 OR #37 OR #38 OR #39 OR #40
42. #34 AND #41
43. Tobacco
44. Cigarette
45. Cigar*
46. #43 OR #44 OR #45
47. Use
48. Smok*
49. #47 OR #48
50. #46 AND #49
51. Alcohol
52. Ethanol
53. #51 OR #52
54. Use
55. Drink*
56. Consumption*
57. Intake
58. Habit
59. #54 OR #55 OR #56 OR #57 OR #58
60. #53 AND #59
61. Diet
62. "Unhealthy diet"
63. Nutrition
64. Dietary
65. "Food habit"
66. Salt
67. Sodium
68. Fruit*
69. Vegetable*
70. Exercise
71. Inactivity
72. "Insufficient activity"
73. "Insufficient exercise"
74. Sedentary
75. "Physical activity"
76. "Physical inactivity"
77. #61 OR #62 OR #63 OR #64 OR #65 OR #66 OR #67 OR #68 OR #69 OR #70 OR #71 OR #72 OR #73 OR #74 OR #75 OR #76
78. Africa South of the Sahara [MeSH]
79. Angola
80. Benin
81. Botswana
82. "Burkina Faso"
83. Burundi
84. Cameroon
85. "Cabo Verde"
86. "Central African Republic"
87. Chad
88. Comoros
89. Congo
90. "Cote d’Ivoire"
91. "Ivory Coast"
92. Djibouti
93. "Equatorial Guinea"
94. Eritrea
95. Eswatini
96. Swaziland
97. Ethiopia
98. Gabon
99. Gambia
100. Ghana
101. Guinea
102. Guinea-Bissau
103. Kenya
104. Lesotho
105. Liberia
106. Madagascar
107. Malawi
108. Mali
109. Mauritania
110. Mauritius
111. Mozambique
112. Namibia
113. Niger
114. Nigeria
115. Rwanda
116. "Sao Tome and Principe"
117. Senegal
118. Seychelles
119. "Sierra Leone"
120. Somalia
121. "South Africa"
122. "South Sudan"
123. Sudan
124. Tanzania
125. Togo
126. Uganda
127. Zambia
128. Zimbabwe
129. #78 OR #79 OR #80 OR #81 OR #82 OR #83 OR #84 OR #85 OR #86 OR #87 OR #88 OR #89 OR #90 OR #91 OR #92 OR #93 OR #94 OR #95 OR #96 OR #97 OR #98 OR #99 OR #100 OR #101 OR #102 OR #103 OR #104 OR #105 OR #106 OR #107 OR #108 OR #109 OR #110 OR #111 OR #112 OR #113 OR #114 OR #115 OR #116 OR #117 OR #118 OR #119 OR #120 OR #121 OR #122 OR #123 OR #124 OR #125 OR #126 OR #127 OR #128
130. #42 OR #50 OR #60 OR #77 OR #129
